# Analysis of the Influence of the Breaking Radiation Magnetic Field of a 10 kV Intelligent Circuit Breaker on an Electronic Transformer

**DOI:** 10.3390/s21237800

**Published:** 2021-11-24

**Authors:** Wenchao Lu, Jiandong Duan, Lin Cheng, Jiangping Lu, Xiaotong Du

**Affiliations:** 1 School of Electrical Engineering, Xi’an University of Technology, Xi’an 710048, China; duanjd@xaut.edu.cn (J.D.); 2190320043@stu.xaut.edu.cn (X.D.); 2 Electric Power Research Institute of State Grid Shaanxi Electric Power Company, Xi’an 710100, China; Chaplin138@163.com (L.C.); qq365323795@163.com (J.L.)

**Keywords:** primary and secondary integration, intelligent switch, electromagnetic interference, ball gap, electronic transformer

## Abstract

The development of the smart grid requires the distribution switch to not be limited to the original breaking function. More functional requirements lead to more complex switch structures, especially the intelligent processing unit on the secondary side. A technology called primary and secondary integration optimizes the structure of the switch, which greatly increases the intelligence level of the switch, but also has disadvantages. The secondary intelligent unit is arranged close to the primary high-voltage electromagnetic environment, and the distribution switch is prone to failure due to electromagnetic interference. In order to explore the influence of electromagnetic interference on it, a transient electromagnetic interference simulation test platform was built for a 10 kV intelligent distribution switch based on the principle of spherical gap arc discharge, and the interference signal of the intelligent distribution switch was measured; the law of the spatial magnetic field near the electronic transformer is mainly studied in this paper. The shielding effectiveness of the distribution terminal of the switch was analyzed, and the interference of the power line of the sensor merging unit circuit board was calculated. The results show that the electronic transformer may have serious faults under continuous strong transient electromagnetic interference. The electromagnetic transient simulation test system studied in this paper can evaluate the anti strong electromagnetic interference ability of the electronic transformer.

## 1. Introduction

The electronic transformer and other secondary equipment in the primary and secondary integration distribution switch are closer to the high-voltage primary side and become a more integrated and standardized intelligent unit [1,2]. This equipment is an indispensable part of the intelligent development of the power system in the future. With the wide application of a primary and secondary integration intelligent switch in the distribution network and the deepening of integration degree, the problem of electromagnetic interference is becoming more and more serious [3,4,5]. According to statistics, since its formal application, electronic transformer faults caused by electromagnetic interference have occurred in many substations and distribution network stations [6]. However, at present, there are no relevant standards consistent with the actual working conditions on site, and the anti-interference characteristics of the secondary side of the primary and secondary integration distribution switch cannot be determined by corresponding indicators [7]. Therefore, the research on the strong electromagnetic disturbance and its test method of the primary and secondary integration distribution switch has important theoretical and application value.

In recent years, the research of relevant scholars on the electromagnetic interference test method of primary and secondary integration distribution switches mainly focused on the conducted interference; that is, only the interference current or voltage measured on the conductor is analyzed. Such research results make it difficult to distinguish the direct coupling superposition of complex electromagnetic radiation signals in space. Scholars studied the multiple arcing and restriking of the circuit breaker in the miniaturized switchgear, analyzed its electromagnetic compatibility characteristics, and conducted the opening and closing test for a medium voltage switchgear to explore its interference source. It is considered that the opening and closing of the disconnector causes more interference to the intelligent power terminal (IED) than the opening and closing of the circuit breaker [8]. One paper [9] compared the IEC 61000 standard with measuring the distribution of power frequency (50/60 Hz) steady-state electromagnetic field of a medium voltage switchgear on all visible conductors of the equipment, and proposed a design safety distance. According to the research in [10], the electromagnetic interference of arc combustion on the secondary circuit of the switch is very serious. Relevant scholars have also conducted electromagnetic interference tests on some intelligent components of the substation, but most of them only measured the electromagnetic field intensity of the nearby space [11,12]. Therefore, some researchers put forward the immunity test method of the electronic transformer, using the disconnector opening and closing to simulate interference source [13], which showed that the electronic transformer was strongly disturbed in the test process. In [14,15], the output of the current sensor and the input and output current signals of control unit during the opening and closing of distribution switch and switchgear were analyzed, respectively, and the main frequency distribution of electromagnetic interference was obtained. In [16], the conducted interference signals at the secondary side of the sensor and the input of the feeder terminal unit (FTU) were measured, and the anti-lightning impulse circuit of the primary and secondary integration distribution switch was optimized. In [17,18], the spatial magnetic field generated by the inrush current when the circuit breaker is put into the capacitor bank was measured, and the main distribution range of electromagnetic pulse was obtained. At present, the research on the electromagnetic radiation interference of the primary and secondary distribution switches is relatively limited, and the radiation propagation path, influence mode, and degree are still unclear.

Based on the problems involved in the above research, the action object and consequences of radiation interference are neglected in much research. Therefore, it is urgent to study the interference of the primary and secondary integration switch electronic transformer under the radiated electromagnetic field. The main contributions of this paper are summarized as follows.

Firstly, a simulation test platform based on the arc discharge principle is established to discharge the primary and secondary integration distribution switch by controlling the ball gap breakdown distance. The spatial magnetic field near the switch sensor of the test object is measured, and the three-dimensional magnetic field signal is verified by the finite integral time domain (FITD) method of an electromagnetic field. The secondary circuit is modeled under the radiation signal, and the shielding efficiency of the shield of the secondary intelligent component and the differential mode electromagnetic interference signal of the merging unit of the sensor core component are calculated. Through this study, the analysis of the disturbance of the electronic transformer on the distribution switch and further research on protection technology are promoted.

## 2. Test Platform

### 2.1. Structure of Tested Intelligent Switch

**Figure 1:**
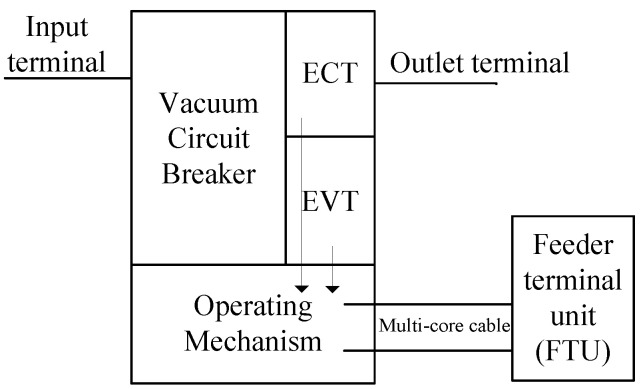
Structure diagram of primary and secondary integration distribution switch.

The structural diagram of the primary and secondary integration distribution switch is shown in Figure 1. Compared with the traditional distribution switch, the tested switch adopts the combination of an electronic current sensor and an electronic voltage sensor, and the current and voltage sensors are integrated with the circuit breaker. The tested distribution switch is equipped with a split phase sensor and feeder terminal unit (FTU). The current sensor is installed in parallel at the switch outlet.

**Figure 2:**
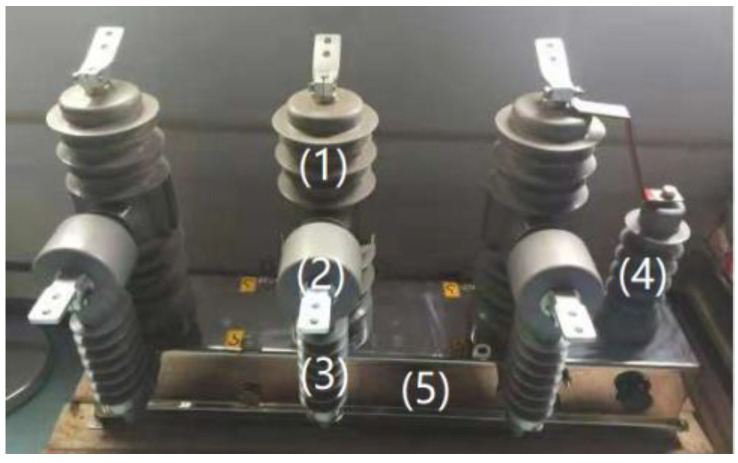
ZW32E−12 distribution switch circuit break.

Figure 2 is the physical diagram of the ZW32E-12 outdoor high voltage vacuum distribution switch of the test object: (1) vacuum circuit breaker; (2) low power electronic current transformer, transformation ratio 600 A:1 V; (3) capacitive voltage divider electronic voltage sensor, transformation ratio 10 kV:3.25 V; (4) capacitor power taking device; and (5) mechanical spring operating mechanism.

### 2.2. Test Principle and Method

**Figure 3:**
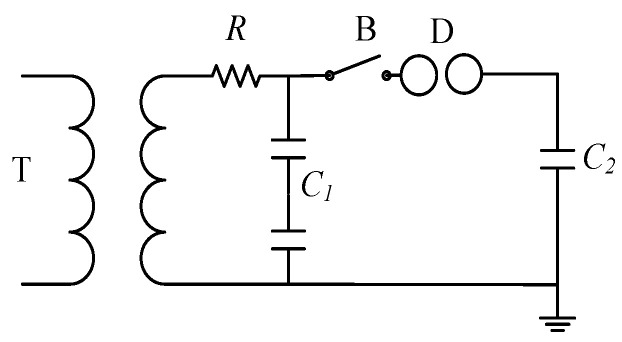
Schematic diagram of test circuit.

The test circuit is shown in Figure 3. Due to its strong controllability and small dispersion of interference signal, this method is the mainstream test method for transient interference of an electronic transformer. This research created a simplified design. T is the test transformer, which is used to provide power for the test circuit; R is the protection resistance of the test transformer; C_1_ is the power side capacitance, which is used to simulate the distributed capacitance (5 nF) on the switching power side in the actual system; and B is the switch under test, and its over opening and closing control the generation of electromagnetic pulse in the process of ball gap arc. The opening and closing interval is the duration of transient electromagnetic disturbance; D is the tested ball gap; and C_2_ is the load side capacitance (3300 pF), which is used to provide electromagnetic energy for the transient process of the test system.

**Figure 4:**
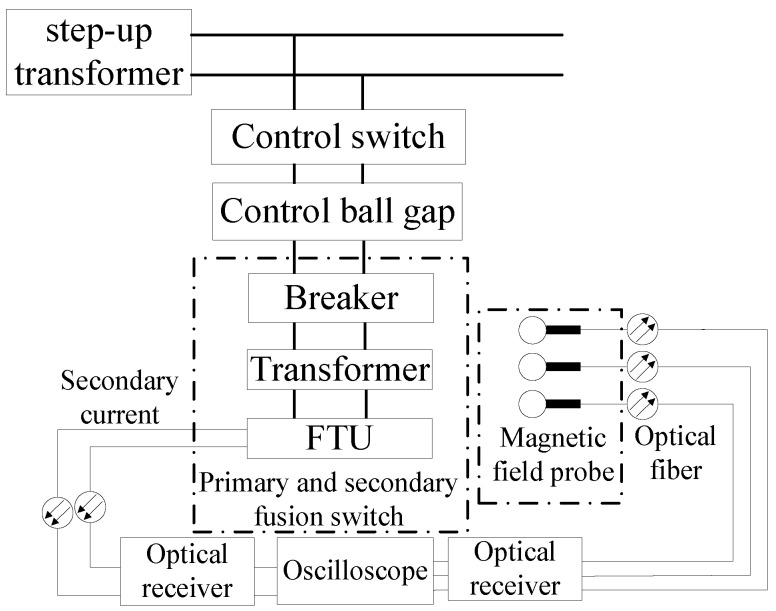
Test wiring diagram.

The test wiring diagram is shown in Figure 4. The optical fiber measuring device was used to measure the secondary current and spatial magnetic field signal of the electronic transformer, and the PicoScope 6404 d oscilloscope was used as the recording device. In order to eliminate the influence of conducted interference on transient current measurement and restore the primary current as much as possible, the secondary cable of the transformer was not connected with FTU, but directly led out the measurement point from the current terminal in the cable and adopted optical fiber transmission.

**Figure 5:**
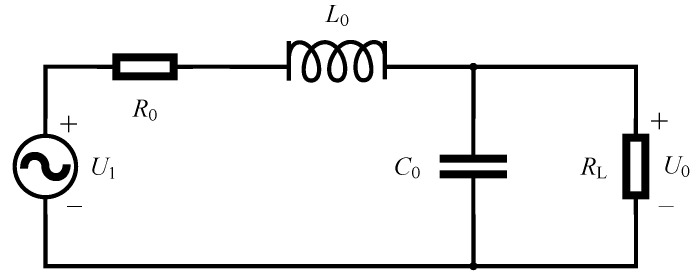
B-dot antenna equivalent circuit.

The B-dot antenna was used to measure the magnetic field in this paper; it was essentially a shielded ring antenna. When the spatial magnetic field changed, resulting in the change in the magnetic flux through the ring antenna, the electromotive force was induced on the ring. Within the turning frequency, this electromotive force was directly proportional to the change rate of the magnetic flux. Its principle is shown in Figure 5.

As shown in Figure 5, the induced electromotive force introduced by the magnetic field is *U*_1_ and the output voltage is *U*_0_. *L*_0_, *C*_0_, and *R*_0_ are the inductance, capacitance, and resistance of the ring antenna, respectively. *R*_L_ is the load impedance. Through derivation, the transfer function of B-dot coil can be simplified as follows.

(1) $$H(s)=\frac{U_{0}(s)}{U_{1}(s)}=\frac{1}{\frac{L_{0}}{R_{L}}s+1}$$

The 3 dB turning frequency *f* of the ring antenna can be expressed by the following Formula (2).

(2) $$f=\frac{R_{L}}{2\pi L_{0}}$$

The frequency characteristics of the antenna are closely related to its size. In this paper, the antenna radius was 2 cm, the antenna conductor radius was 1.1 mm, and the load impedance was 50 Ω. Through theoretical calculation, the turning frequency of 3 dB can reach 106.26 MHz. After simulation, the turning frequency was about 95.99 MHz, which met the test requirements. Changing the antenna size can improve its measurement frequency.

**Figure 6:**
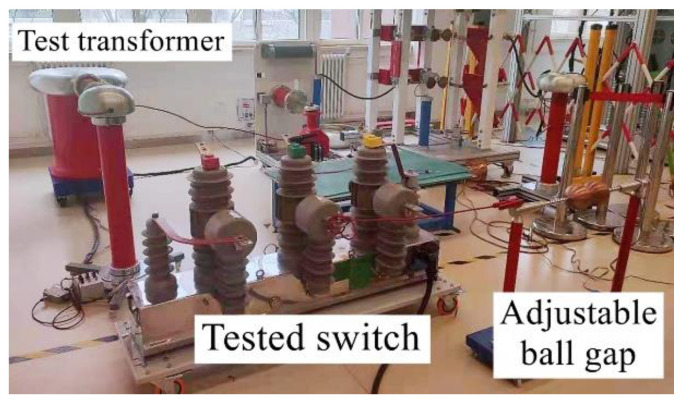
Field wiring diagram.

The actual field wiring is shown in Figure 6. To perform the test, before the test, control and adjust the ball gap spacing to 1 mm, and disconnect the tested switch. After the beginning of test, close the tested switch, and then gradually increase the voltage at both ends of the ball gap by using the step-up transformer. When the ball gap breaks down, record the voltage on the high voltage side of the step-up transformer. When the ball gap discharge time reaches the preset value, separate the tested switch and the test is complete. Collect the output transient current and magnetic field strength of the secondary side of the current sensor. Then, adjust the ball gap spacing to 2 mm and 3 mm, and repeat the previous test steps. Perform 10 tests at each clearance distance.

**Figure 7:**
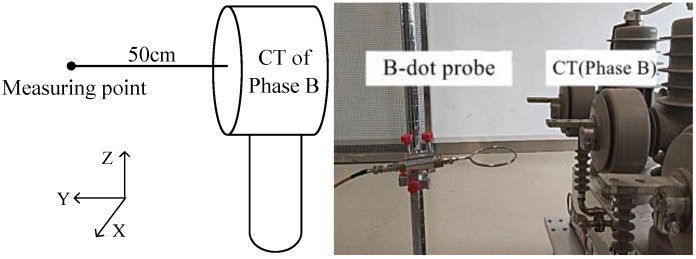
Schematic diagram of magnetic field measurement position.

Considering the safe insulation distance and the anti-interference performance of the measuring port, the magnetic field probe should not be too close to the outgoing terminal of the switch. As shown in Figure 7, the (X,Z) coordinates of the measuring point coincide with the center of phase B electronic current transformer (CT). Considering the safe insulation distance between the probe and the metal conductor and the measurement accuracy, the horizontal distance in Y direction is 50 cm.

## 3. Analysis of Test Results

**Figure 8:**
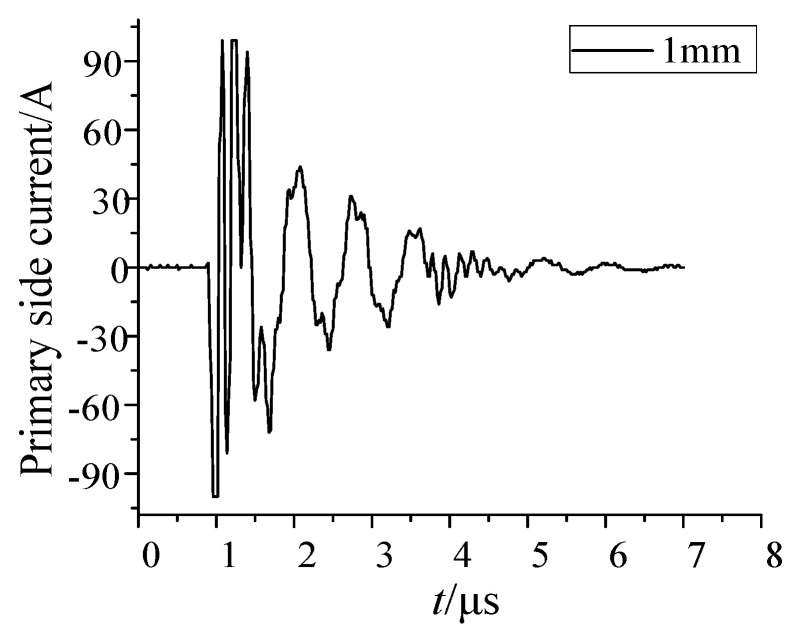
Current at secondary side of sensor (1 mm ball gap distance).

**Figure 9:**
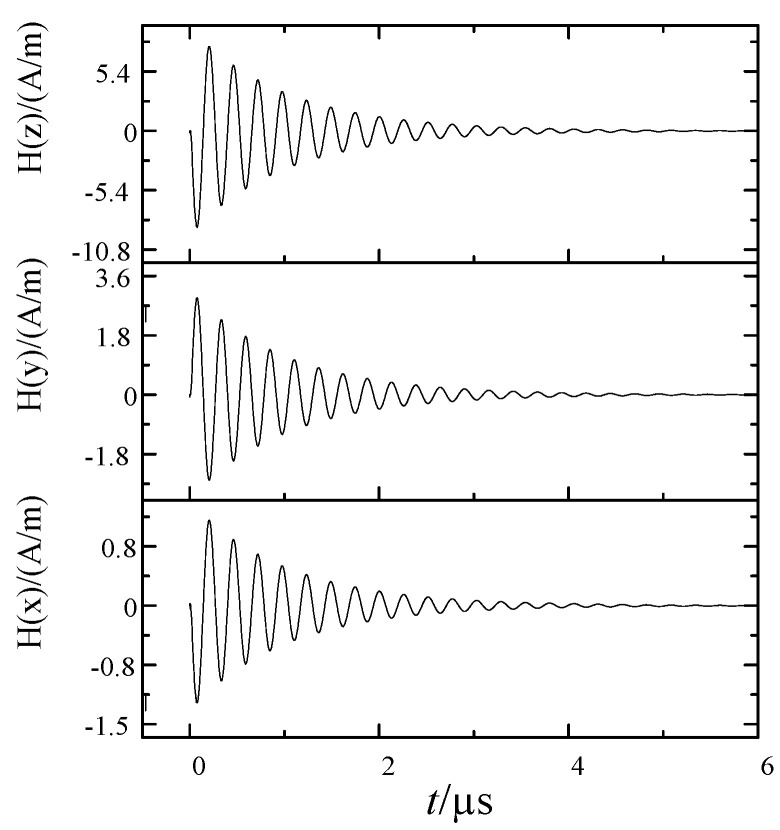
Three dimensional magnetic field waveform (1 mm ball gap).

When the voltage at both ends of the ball gap fracture reached the breakdown voltage, the fracture broke down and formed a conductive arc gap. The voltage difference between the fractures was almost zero, and the electromagnetic interference pulse reached the peak. Then the arc went out, and reburning occurred many times. The current of the current transformer with single breakdown and the three-dimensional magnetic field waveform of measuring point are shown in Figure 8 and Figure 9.

See Table 1 for the average breakdown voltage recorded at the measuring end of the step-up transformer and the relationship between the converted primary current amplitude and the ball gap spacing. Compared with the statistical results of the magnetic field waveform and breakdown voltage, the synthetic magnetic field intensity of the measuring point increased with the increase in ball gap distance.

## 4. Electromagnetic Field Simulation and Result Comparison

In this paper, the interference source of the primary and secondary integration switch electronic transformer was mainly the interference signal generated by ball gap breakdown. The signal traveled outward along the wire. The wire carrying high-frequency signal formed an antenna and radiated around, which can affect the normal operation of secondary equipment [19].

In order to verify the effectiveness of the test and the accuracy of the measurement, the space radiated magnetic field was simulated and verified in this paper. The main frequency of the interference source as excitation was about 1 MHz. It can be considered that the opening and closing interference of analog distribution switch belongs to a small-scale near-field problem. Therefore, this paper considered that the electric field of the measuring point in the space of distribution switch was related to voltage, and the magnetic field was related to current. Based on the above analysis, the time domain calculation method was used [20].

### 4.1. Finite Integral Time Domain Method

Directly using the time domain method to calculate the electromagnetic field can obtain the time domain response at one time, which greatly improves the calculation efficiency. In the finite integral time domain method (FITD), for isotropic linear media, the relationship between magnetic field strength $\vec{H}$ and magnetic flux $\vec{B}$ is as follows [21]:

(3) $$\vec{B}=\mu \vec{H}$$

where *μ* is the permeability of the medium.

In electromagnetic simulation, the magnetic flux $\vec{B}$ of the grid plane is distributed on the main grid, while the magnetic field intensity $\vec{H}$ is defined on the sub grid, and each vertex of the sub grid is defined in the range of the main grid.

(4) $$\overset{⌢}{h}=\int_{{\overset{⌢}{l}}_{i}}H\cdot dl$$

where $\overset{⌢}{h}$ is the defined magnetic pressure, *li* is the edge of the grid, and *l* is the integral loop.

Therefore, the integral expression of Equation (4) can be transformed into grid Equation (5).

(5) $$\sum_{k}{\tilde{C}}_{ik}{\overset{⌢}{h}}_{k}=\frac{d}{dt}\overset{⌢}{\overset{⌢}{d}}+\overset{⌢}{\overset{⌢}{j}}$$

Among these, ${\tilde{C}}_{ik}$ is the spinor operator corresponding to the subnet, ${\overset{⌢}{h}}_{k}$ is the magnetic pressure of the corresponding network, $\overset{⌢}{\overset{⌢}{d}}$ is the electric flux, and $\overset{⌢}{\overset{⌢}{j}}$ is the current operator.

### 4.2. Simulation Calculation of Radiated Magnetic Field

**Figure 10:**
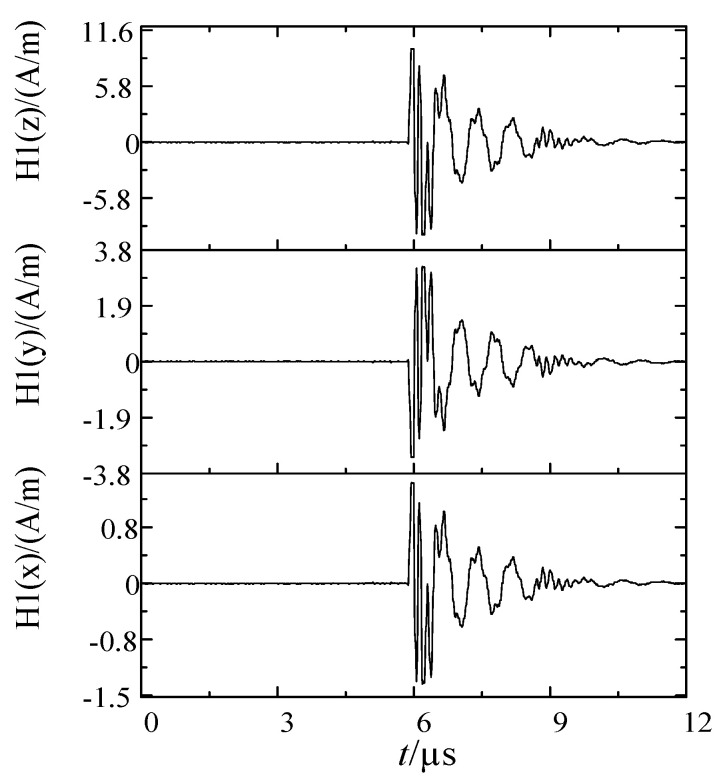
Simulation of the three-dimensional magnetic field waveform of B-phase sensor side (1 mm).

The electromagnetic model of primary and secondary integration distribution switch is established by simulation software. The excitation signal collects the current according to the secondary side of the electronic transformer in the test, and let the current flow through the B-phase conductor in the model. During the simulation, the parameters are set according to the test environment. Set the field monitor according to the actual probe position in the accessories of phase B sensor. Figure 10 shows the calculation results of the three-dimensional magnetic field under the 1 mm ball gap breakdown transient current.

As can be seen from Figure 9, the magnetic field signals in all directions had a higher fitting degree in amplitude and frequency than the measured data. The magnetic field intensity in the Y and Z directions measured at the side of the electronic transformer was relatively large, which was caused by the outgoing terminal near the current transformer parallel to the X direction of the calculation space. It can be considered that Y and Z were the main polarization directions of the measuring point.

The three-dimensional magnetic field of the measuring point is synthesized by Equation (6) [22].

(6) $$H_{Abs}=\sqrt{H_{x}{}^{2}+H_{y}{}^{2}+H_{z}{}^{2}}$$

**Figure 11:**
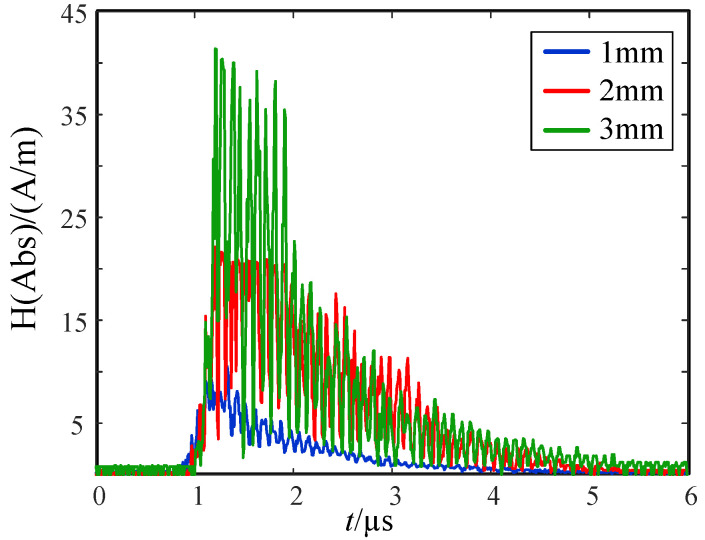
Synthetic magnetic field strength.

Then, the magnetic field data in three directions were synthesized by using the principle of three-dimensional magnetic field synthesis; that is, the magnetic field intensity in the main polarization direction was obtained. Using the same method, the measured 2 mm and 3 mm spherical gap discharge current signals were introduced into the model for calculation, and the obtained synthetic magnetic field is shown in Figure 11.

**Figure 12:**
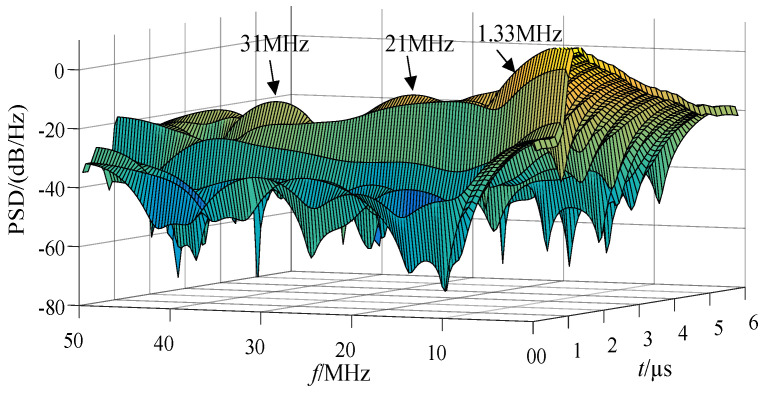
Power spectrum of simulated magnetic field intensity (1 mm).

The three-dimensional time-frequency analysis was performed on the synthetic magnetic field waveform in Figure 11 to obtain the power spectral density (PSD) diagram, shown in Figure 12.

**Figure 13:**
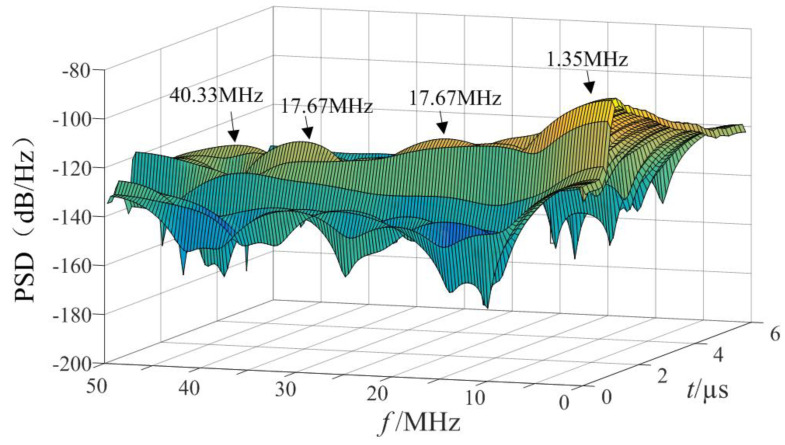
Power spectrum of simulated magnetic field intensity (2 mm).

**Figure 14:**
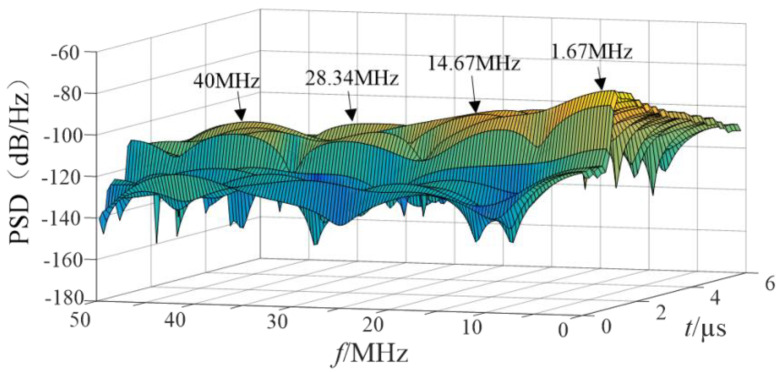
Power spectrum of simulated magnetic field intensity (3 mm).

According to the scale positioning of the peak wave head in Figure 12, the characteristic frequencies were about 1.33 MHz, 21 MHz, 31 MHz, etc., and the energy was high at the characteristic frequency. The high-frequency component decayed quickly, the duration was short, the PSD value at the characteristic frequency was small, and the energy was weak. Figure 13 and Figure 14 shows the PSD of the 2 mm and 3 mm ball gap, which had similar spectral characteristics.

## 5. Analysis of Influence of Radiated Magnetic Field on Intelligent Unit

The space radiated magnetic field generated by the transient current signal during ball gap breakdown will inevitably affect the merging unit of electronic transformer, but whether it will affect the normal operation of the sensor or even destroy the device is difficult to measure only by the strength of the space magnetic field [23]. This paper further studies and analyzes the intelligent component used in the engineering application of the tested primary and secondary integration switch.

### 5.1. Shielding Effectiveness Analysis of Fedder Terminal Unit (FTU)

Shielding effectiveness refers to the catadioptric reflection of the metal conductor on the incident interference signal, and finally reduction in the interference component received by the protected part of the metal conductor. The *SE_H_* expression of magnetic field shielding effectiveness is defined as [24]:

(7) $$SE_{H}=20\mathrm{lg}(H_{1}/H_{2})$$

where *H*_1_ is the magnetic field strength before shielding, and *H*_2_ is the magnetic field strength after shielding.

**Figure 15:**
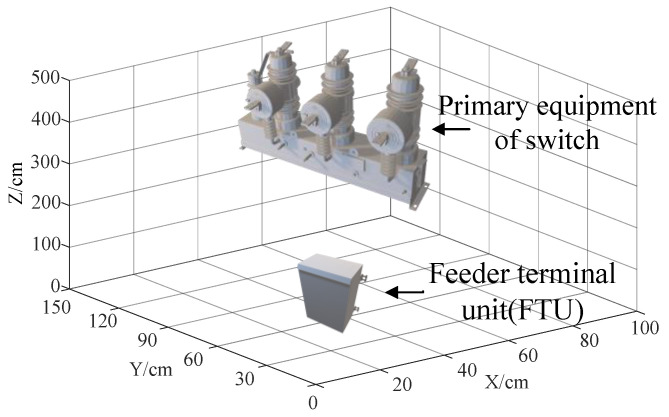
Simulation model of distribution switch with FTU.

At present, intelligent units in the project are generally installed inside the FTU. According to the field installation standard, the FTU is usually installed with the switch on the column, and the clearance distance from the switch is not less than 2 m [25]. In this paper, the installation distance between the switch and FTU was divided into 1 m (meter), 3 m, 6 m, and 9 m according to the actual cable length. Considering the actual tower height and the worst interference situation, we selected the solid modeling of FTU at 1 m below the switch on the column. The box size was 50 × 30 × 20 cm and 2 mm thick. The specific layout structure is shown in Figure 15.

**Figure 16:**
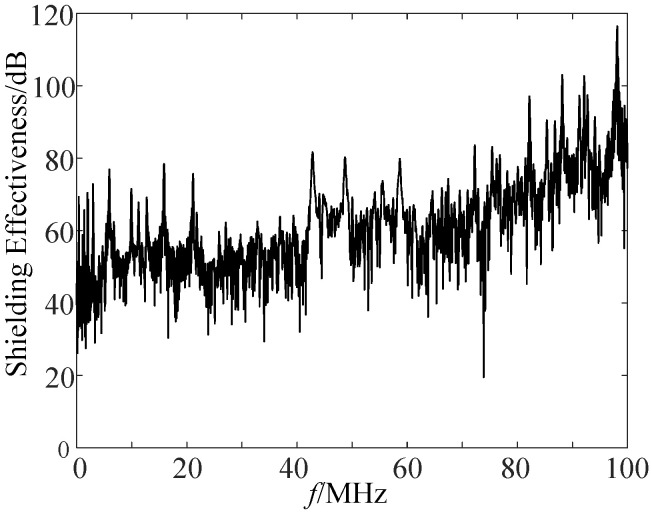
Shielding effectiveness of the FTU shell.

The measuring point for calculating shielding effectiveness is located at the geometric center of the corresponding position of FTU, which is equivalent to the position of the sensor merging unit board in the real object. When calculating, we set the frequency as 10–100 MHz and took the 1 mm ball gap breakdown current as the excitation to obtain its shielding efficiency, as shown in Figure 16.

We took more than 60 dB as the excellent shielding efficiency range. According to the results in Figure 16, the primary and secondary integration distribution switch had good shielding efficiency for the magnetic field of radiated signals with a frequency of 78 MHz to 100 MHz. The highest shielding efficiency could reach 118.5 dB, and the shielding efficiency fluctuated greatly for signals below 78 MHz. According to the spectrum analysis results in Figure 11, the interference signal energy was mainly distributed at about 1 MHz to 30 MHz, it did not belong to the good shielding interval. Therefore, the shielding body used by the existing engineering equipment cannot achieve the ideal protection effect on the interference signal.

### 5.2. Differential Mode Interference Calculation of Combined Unit

**Figure 17:**
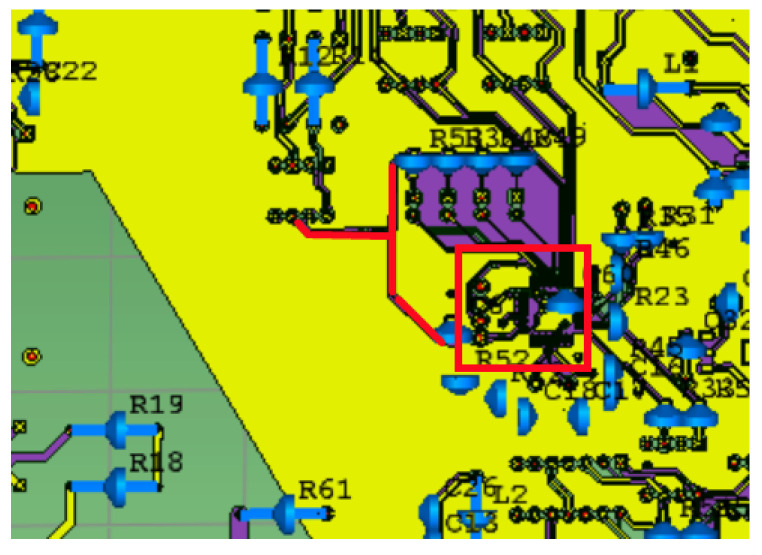
Local model of merging unit.

The general structure of the electronic transformer includes primary conversion, secondary conversion, and a merging unit. Among these, the merging unit is located in three parts of the FTU box, which undertakes the functions of transformer signal acquisition and processing. It is the central structure of the electronic transformer. However, in practical application, the faults of the merging unit caused by electromagnetic interference account for a large proportion. Most of the merging units used in practical engineering are FPGA core processors (red box in Figure 17), and the routing of analog and digital signals is complex. The radiation signal enters through the slot of the FTU shell, which may affect the signal acquisition of the merging unit. In this paper, the combined unit board of a mainstream primary and secondary integration switch product was selected for three-dimensional modeling, and the chip driving power was provided by the power supply module with 3.3 V (Red line segment in Figure 17).

**Figure 18:**
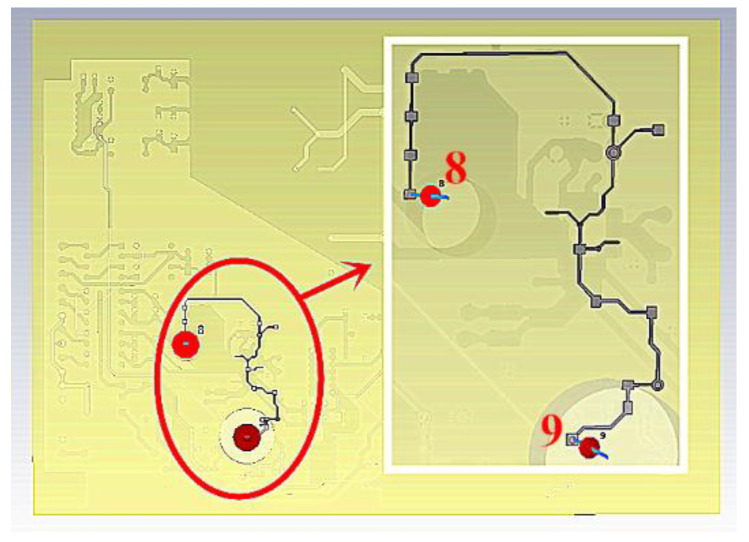
Schematic diagram of the 3.3 V power line.

The position of 3.3 V voltage in the board is shown in Figure 18. During simulation, we injected 3.3 V voltage into the line, of which 8 was the injection end, 9 was the output end, the port impedance was 50 Ω, and the calculation mode of the port was differential mode.

**Figure 19:**
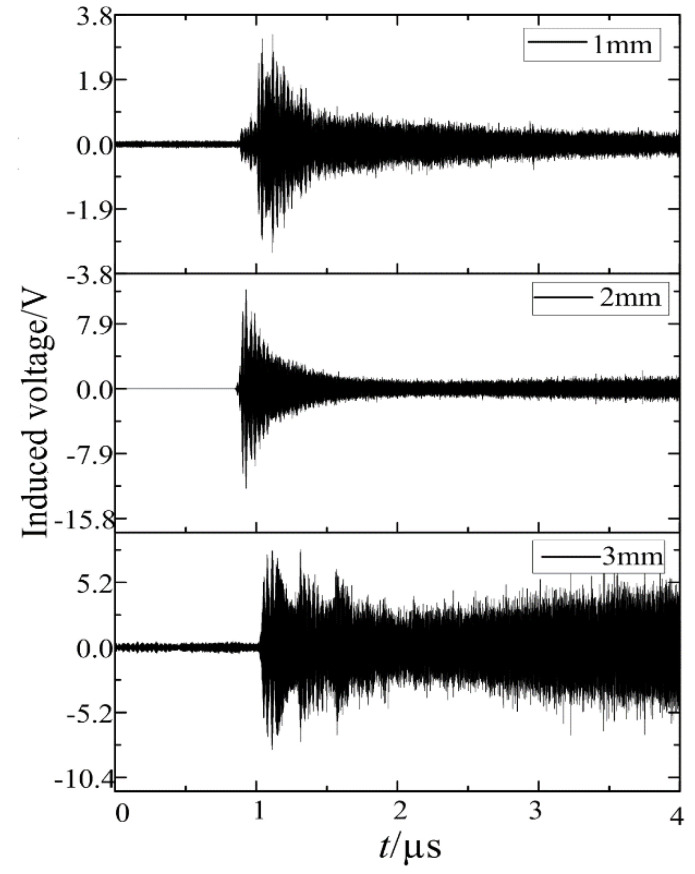
Induced voltage of the 3.3 V power line.

The circuit breaker, FTU shell, and intelligent component were simulated according to the actual installation position. The interference signal in the simulation used the ball gap breakdown current at the spacing of 1 mm, 2 mm, and 3 mm. The acquisition frequency band was 10 Hz–100 MHz. After that, the voltage waveform on the 3.3 V voltage line could be obtained. Figure 19 shows the voltage waveform of 3.3 V port under different ball gap spacing. The peak induced voltage could reach 3.26 V, 6.14 V, and 7.62 V, respectively. Referring to the common chip manual of distribution switch and relevant standards, the maximum rated voltage of a chip 3.3 V power line is between 5 V and 7.5 V. According to the above simulation disturbance test platform, when the strong electromagnetic interference assessment of electronic transformer was carried out, the duration included dozens of pulses. If affected by the load, the arc may continue to burn and cannot be extinguished. Under the direct coupling electromagnetic disturbance, it is likely to cause measurement error or damage the chip.

**Figure 20:**
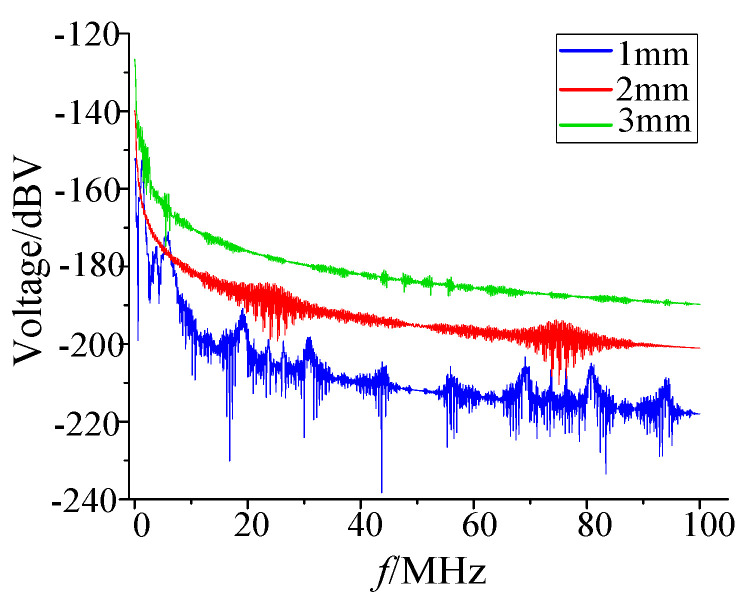
Frequency domain distribution of induction voltage in the 3.3 V power line.

The interference signal was decomposed in the frequency domain (Figure 20). The frequency band distribution was roughly the same under different gaps, and the characteristic frequencies were about 5 MHz, 10 MHz, 43 MHz, 16.8 MHz, and 80 MHz. Compared with the power spectral density distribution of the pulse current, its high-frequency component was more abundant.

## 6. Conclusions

In this paper, the radiated magnetic field interference of the electronic transformer of the primary and secondary integration distribution switch was studied, the space magnetic field was measured and verified through test and simulation, and the shielding efficiency and coupling interference were calculated and analyzed for the actual intelligent components of the electronic current transformer. The main conclusions are as follows:

(1) It is feasible and effective to evaluate the anti strong electromagnetic disturbance ability of the electronic transformer in the primary and secondary integration switch by using the ball gap discharge simulation electromagnetic disturbance test;
(2) The simulated electromagnetic disturbance test method can simulate the arc short-circuit fault in the distribution network system, which is equivalent and consistent with the actual working conditions on site. Under the action of strong electromagnetic disturbance, the magnetic field intensity near the electronic transformer exceeded the existing standard range, and the maximum magnetic field intensity could reach 50 A/m; in terms of frequency, high frequency components above 1 MHz were abundant. There were many high-frequency components above 30 MHz, and the source of these components is worthy of further research and exploration;
(3) The shielding effectiveness of the protective body of the merging unit of the electronic transformer was analyzed. Its shielding effect had limitations, and the shielding effectiveness below 78 MHz was relatively weak; the space radiated magnetic field produced a differential mode voltage of up to 7.62 V on the power line of the merging unit. This interference is likely to lead to the measurement error of the electronic transformer, and the chip may be damaged under repeated similar interference.

In the future, there is much work to be further conducted, such as the actual measurement of the voltage and current on the electronic board, the electromagnetic field measurement test inside the shielding shell, and improving the measurement range and accuracy of the magnetic field antenna. After some new energy sources are connected (such as wind power, electric vehicles, etc.), the interference signal may have new characteristics, and so on.

## Figures and Tables

**Table 1:** Relationship between gap spacing and breakdown voltage.

Distance of Ball Gap/mm	Average Breakdown Voltage/kV	Discharge Current Amplitude/A
1	2.44	95–102
2	4.64	125–132
3	7.62	176–216

## Data Availability

Not involved.
